# The Predictive Roles of Tumour Markers, Hemostasis Assessment, and Inflammation in the Early Detection and Prognosis of Gallbladder Adenocarcinoma and Metaplasia: A Clinical Study

**DOI:** 10.3390/ijms26083665

**Published:** 2025-04-12

**Authors:** Andrei Bojan, Catalin Pricop, Maria-Cristina Vladeanu, Iris Bararu-Bojan, Codruta Olimpiada Halitchi, Simona Eliza Giusca, Oana Viola Badulescu, Manuela Ciocoiu, Dan Iliescu-Halitchi, Liliana Georgeta Foia

**Affiliations:** 1 Department of Surgical Specialties I, Faculty of Medicine, Grigore T. Popa University of Medicine and Pharmacy, 16 Universitatii Street, 700111 Iasi, Romania; andrei.bojan@yahoo.com (A.B.);; 2 Department of Morpho Functional Sciences, Faculty of Medicine, Grigore T. Popa University of Medicine and Pharmacy, 16 Universitatii Street, 700111 Iasi, Romaniamanuela.ciocoiu@umfiasi.ro (M.C.); 3 Department of Pediatry, Faculty of Medicine, Grigore T. Popa University of Medicine and Pharmacy, 16 Universitatii Street, 700111 Iasi, Romania; 4 Department of Medical Sciences, Faculty of Medicine, Grigore T. Popa University of Medicine and Pharmacy, 16 Universitatii Street, 700111 Iasi, Romania; 5 Discipline of Biochemistry, Faculty of Dental Medicine, Grigore T. Popa University of Medicine and Pharmacy, 16 Universitatii Street, 700115 Iasi, Romania

**Keywords:** hemostatic dysregulation, gallbladder metaplasia, chronic inflammation, gallbladder cancer

## Abstract

Gallbladder carcinoma (GBC) is one of the most aggressive malignancies of the biliary tract, often originating from chronic inflammation associated with gallstones and cholecystitis. Persistent inflammation plays a pivotal role in the development of preneoplastic changes, such as metaplasia, which may progress to malignancy. Despite its relatively low incidence, GBC is characterized by a poor prognosis due to late-stage diagnosis, highlighting the urgent need for improved early detection strategies. This study aimed to assess the diagnostic and prognostic significance of CA 19-9 and CEA levels in patients with gallbladder lesions, while also evaluating systemic inflammation and hemostatic dysregulation. A retrospective analysis was conducted on patients diagnosed with gallbladder lesions, with histopathological confirmation of adenocarcinoma and metaplasia. Laboratory assessments included serum levels of tumour markers, inflammatory markers such as CRP, and key hemostatic parameters, including thrombocyte count, prothrombin time (PT), activated partial thromboplastin time (aPTT), and fibrinogen levels. A predictive scoring model was developed using the cutoff values of CA 19-9 and CEA to assess their combined diagnostic potential. Among the patients studied, 48.9% had an initial diagnosis of chronic cholecystitis, while 32.2% presented with acute cholecystitis. Adenocarcinoma was identified in 6.7% of cases after histopathological examination, predominantly in females over 65 years old with acute cholecystitis. Metaplasia was detected in 7.8% of cases, primarily in elderly females with chronic cholecystitis. Laboratory findings revealed significantly elevated levels of CA 19-9, CEA, AFP, and CA-125 in patients with adenocarcinoma. Additionally, abnormalities in hemostatic parameters, including increased fibrinogen levels and alterations in thrombocyte count, were observed in patients with malignancy. A combined predictive score using CA 19-9 and CEA demonstrated strong potential for detecting adenocarcinoma and metaplasia, improving diagnostic accuracy. Our findings emphasize the clinical importance of integrating tumour markers, inflammatory biomarkers, and hemostatic parameters in the evaluation of gallbladder lesions associated with chronic inflammation. The combined assessment of these factors enhances early detection, facilitates malignancy risk stratification, and improves prognostic evaluation, particularly in patients with metabolic and cardiovascular comorbidities.

## 1. Introduction

The gallbladder is susceptible to various diseases, ranging from inflammation and preneoplastic conditions to malignant tumours. One of the most aggressive forms is gallbladder carcinoma (GBC), which is known to arise from chronic gallbladder inflammation that can lead to preneoplastic changes and, eventually, malignancy. Although GBC has a relatively low incidence, its prognosis can be significantly improved with early detection. Gallbladder cancer (GBC), although rare, is one of the most aggressive malignancies in the digestive system, with the incidence varying significantly across different geographic regions. It is more common in countries such as India, Japan, and certain regions of Latin America, where it ranks among the leading causes of cancer-related mortality. In contrast, its incidence is relatively low in Western populations. According to recent epidemiological studies, the global age-standardized incidence rate of gallbladder cancer ranges between 0.5 and 3.0 per 100,000 individuals, with a higher incidence in women, particularly those over the age of 65. Furthermore, the prevalence of gallbladder lesions, including both benign and malignant conditions, has been increasing due to advancements in diagnostic imaging and the growing number of risk factors such as obesity, diabetes, and gallstones. Chronic cholecystitis is often cited as a precursor to both benign and malignant gallbladder lesions, further emphasizing the clinical importance of monitoring patients with a history of chronic gallbladder disease.

Unfortunately, most cases are diagnosed at an advanced stage, making complete surgical removal difficult or even impossible [1,2]. Gallstones and chronic cholecystitis are associated with GBC in 60–90% of cases, while up to one-third of patients with GBC also present with xanthogranulomatous cholecystitis (XGC). Over the past three decades, limited progress has been made in the diagnosis and treatment of GBC, largely due to an incomplete understanding of its molecular mechanisms. While most gallbladder lesions are benign, untreated chronic cholecystitis can lead to premalignant changes that may progress to invasive forms of neoplasia.

Several genetic mutations and alterations have been identified as risk factors for gallbladder neoplasia, including mutations in tumour suppressor genes such as *TP53* and oncogenes like HER-2/neu. These genetic predispositions, coupled with chronic inflammation from conditions such as chronic cholecystitis and cholelithiasis, may lead to the progression of benign gallbladder lesions to more advanced neoplasms. Environmental factors, such as exposure to certain chemicals and toxins, as well as a diet rich in fats and low in fibre, are also considered potential contributors to the development of gallbladder lesions. The role of infections, particularly those caused by *Salmonella* and *Helicobacter pylori*, has also been implicated in gallbladder carcinogenesis. Recent studies have highlighted the significance of precursor lesions and apomucin expression in gallbladder tissue obtained from cholecystectomy specimens, reinforcing the need for early diagnosis [3,4]. In some instances, GBC is incidentally detected during cholecystectomy for gallstones, providing a crucial opportunity for early intervention. However, late-stage diagnoses are often associated with widespread disease, lymph node involvement, and a higher likelihood of recurrence following surgical resection [5,6].

To improve understanding of GBC pathophysiology and tumour development, it is essential to explore metabolic, inflammatory, and environmental factors alongside mucin immunohistochemistry properties. Additionally, investigating genetic abnormalities, such as karyokinetic irregularities in the gallbladder’s proximal mucosa, may yield valuable insights. A deeper understanding of these factors could help to identify individuals at higher risk of developing neoplasia, ultimately leading to better diagnostic, preventive, and therapeutic strategies [7,8].

Tumour markers such as carcinoembryonic antigen (CEA), CA 242, and CA 19-9 are commonly used in cancer detection. Although CEA and CA 19-9 have historically been employed as tumour markers for GBC, their sensitivity is relatively low when used individually. Consequently, relying solely on these markers for diagnosis can yield inconsistent results. However, studies suggest that combining these markers significantly enhances the specificity and sensitivity of GBC diagnosis. Despite this, only a limited number of studies have evaluated the combined use of these markers, emphasizing the need for further research to refine diagnostic accuracy and improve early detection strategies [9,10]. Both of these markers have demonstrated significant potential in detecting gastrointestinal neoplasms, particularly those affecting the gallbladder and biliary system. CEA is widely recognized for its role in diagnosing and monitoring colorectal cancer, but increased levels of CEA have also been found in patients with gallbladder cancer. Similarly, CA 19-9 is known for its utility in detecting pancreatic and biliary tract malignancies, and elevated levels are often seen in gallbladder cancer patients. Given their established relevance in gastrointestinal cancers, we aimed to explore the potential of CEA and CA 19-9 as diagnostic and prognostic tools specifically for gallbladder lesions, especially since early diagnosis remains a major challenge in improving patient outcomes [11].

## 2. Results

A total of 90 patients participated in this study. Among them, 6.7% were diagnosed with adenocarcinoma, while the remaining patients were diagnosed with benign gallbladder disease (GBD) or dysplasia. The median age of the participants was 64.0 years, with an interquartile range spanning from 28 to 85 years. Additionally, the study cohort predominantly comprised women. The most common initial diagnosis among the study participants was chronic cholecystitis (48.9%), followed by acute cholecystitis (32.2%).

Among the patients (48.9%) diagnosed with chronic cholecystitis, 95.5% were female (*p* = 0.001), and 59.1% were under 65 years old (*p* = 0.006). For the patients (32.2%) diagnosed with acute cholecystitis, 72.4% were female (*p* = 0.001), and 72.4% were under 65 years old (*p* = 0.001). Acute pancreatitis was identified in 6.7% of cases, with a predominance of male patients (66.7%) (*p* = 0.006), all over the age of 65 (*p* = 0.006). Acute pyocholecystitis was diagnosed in 5.6% of individuals in the studied group, all of whom were female (*p* = 0.001), with 60% being over the age of 65 (*p* = 0.006).

### 2.1. Adenocarcinoma

Of the 6.7% of the patients who had adenocarcinoma identified as a result of the anatomopathological exam, all cases occurred in female patients (100%; *p* = 0.033) over the age of 65 (100%; *p* = 0.033) and in those with acute cholecystitis (100%; *p* = 0.001). Among the monitored laboratory parameters, patients with adenocarcinoma exhibited significantly higher levels of CA 19-9 (563.69 vs. 41.96 ng/mL; *p* = 0.001), CEA (30.67 vs. 2.81 ng/mL; *p* = 0.001), AFP (13.0 vs. 5.21 ng/mL; *p* = 0.001), and CA-125 (578.27 vs. 44.50 ng/mL; *p* = 0.001), while amylase (46.0 vs. 190.89 U/L; *p* = 0.001) and total bilirubin (0.55 vs. 1.13 mg/dL; *p* = 0.047) levels were significantly lower (see Table 1).

**Figure 1:**
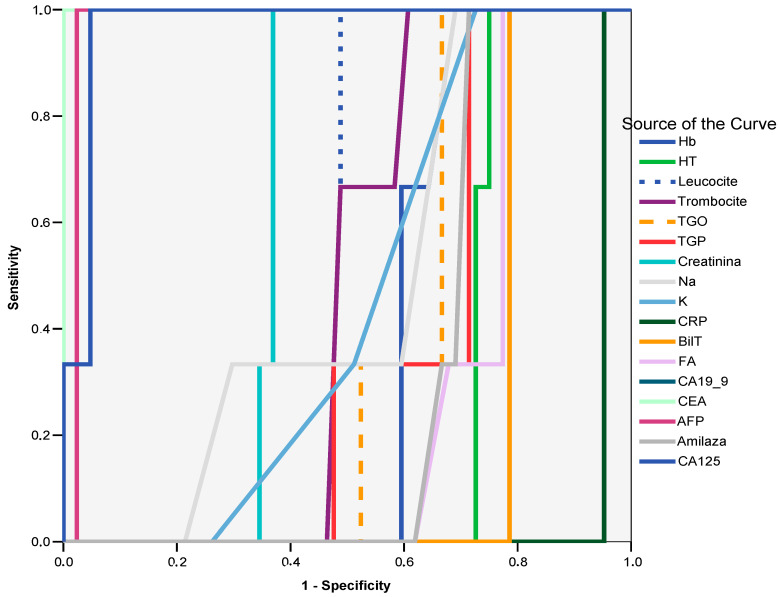
Diagnostic performance of tumour markers in predicting adenocarcinoma: ROC curve analysis. Curve legend: Hb—hemoglobin, HT—hematocrit, Leucocite—leucocytes, Trombocite—thrombocytes, TGO—ASAT, TGP—ALAT, Creatinina—creatinine, BilT—total bilirubin, Amilaza—amylase.

The ROC curve analysis confirmed the following as strong predictors of adenocarcinoma: CEA (AUC = 1.000; 95% CI: 1.0–1.0; *p* = 0.001), with values above 25 ng/mL showing 100% sensitivity and 100% specificity; CA-125 (AUC = 0.968; 95% CI: 0.932–1.005; *p* = 0.001), with values above 319 ng/mL demonstrating 100% sensitivity and 95% specificity; CA 19-9 (AUC = 0.968; 95% CI: 0.932–1.005; *p* = 0.001), with values exceeding 307 ng/mL achieving 100% sensitivity and 95% specificity; and AFP (AUC = 0.976; 95% CI: 0.944–1.009; *p* = 0.001), with values above 12.55 ng/mL providing 100% sensitivity and 97% specificity (Figure 1).

### 2.2. Metaplasia

Histopathological examination revealed that 7.8% of cases were diagnosed with metaplasia, occurring exclusively in female patients (100%; *p* = 0.197), with 71.4% being over 65 years old (*p* = 0.366) and all cases being associated with chronic cholecystitis (100%; *p* = 0.005). The estimated risk of metaplasia was significantly higher in patients with obesity (RR = 12.96; 95% CI: 4.00–21.9; *p* = 0.006) and hypertension (RR = 14.89; 95% CI: 4.72–25.07; *p* = 0.008). Among the laboratory parameters analyzed, patients with metaplasia exhibited significantly elevated creatinine levels (1.26 vs. 0.90 mg/dL; *p* = 0.001) and amylase levels (1273 vs. 89.19 U/L; *p* = 0.001), while hemoglobin levels were significantly lower (11.50 vs. 13.34 g/dL; *p* = 0.019).

**Figure 2:**
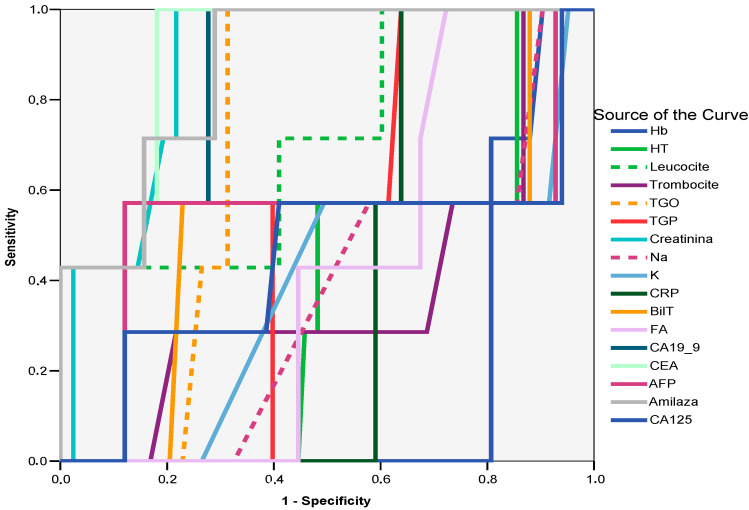
ROC curve. Laboratory parameters predictive of metaplasia. Curve legend: Hb—hemoglobin, HT—hematocrit, Leucocite—leucocytes, Trombocite—thrombocytes, TGO—ASAT, TGP—ALAT, Creatinina—creatinine, BilT—total bilirubin, Amilaza—amylase.

Given these findings, ROC curve analysis was performed to identify strong predictors of metaplasia. The results confirmed that amylase (AUC = 0.873; 95% CI: 0.770–0.975; *p* = 0.001), at values above 59 U/L, demonstrated 100% sensitivity and 71% specificity. Similarly, CEA (AUC = 0.854; 95% CI: 0.778–0.929; *p* = 0.002), with levels greater than 1.46 ng/mL, showed 100% sensitivity and 81% specificity. Additionally, CA 19-9 (AUC = 0.812; 95% CI: 0.719–0.906; *p* = 0.006), at values above 13.57 ng/mL, exhibited 100% sensitivity and 75% specificity, while creatinine (AUC = 0.880; 95% CI: 0.795–0.964; *p* = 0.001), with levels exceeding 1.01 mg/dL, had 100% sensitivity and 78% specificity. These findings underscore the potential of these biomarkers in the early detection and risk stratification of metaplastic changes in gallbladder lesions (see Figure 2, Table 2).

### 2.3. Chronic Colecystitis

Chronic cholecystitis was significantly associated with obesity and hypertension, with the estimated risk being over 9 times higher in obese patients (RR = 9.11; 95% CI: 3.05–27.20; *p* = 0.001) and 4.84 times higher in patients with hypertension (RR = 4.84; 95% CI: 2.42–9.68; *p* = 0.001).

Laboratory analysis revealed that patients with chronic cholecystitis had significantly higher levels of AST (62.85 vs. 23.52 U/L; *p* = 0.011), ALT (102.90 vs. 32.83 U/L; *p* = 0.027), sodium (140.23 vs. 139.04 mg/dL; *p* = 0.041), and amylase (294.61 vs. 72.78 U/L; *p* = 0.041) compared to those without the condition.

ROC curve analysis confirmed AST and ALT as good predictors of chronic cholecystitis. AST levels above 18.60 U/L had a sensitivity of 66% and a specificity of 59%, with an AUC of 0.622 (95% CI: 0.503–0.741; *p* = 0.001). ALT levels above 19.50 U/L showed a sensitivity of 64% and a specificity of 41%, with an AUC of 0.608 (95% CI: 0.466–0.706; *p* = 0.001) (see Table 3).

### 2.4. Acute Cholecystitis

The estimated risk of developing acute cholecystitis was 3.44 times higher in patients with diabetes mellitus (RR = 3.44; 95% CI: 2.47–4.79; *p* = 0.009). Among the monitored laboratory parameters, patients with acute cholecystitis showed significantly higher levels of leukocytes (11,312 vs. 8695 n/µL; *p* = 0.002), alkaline phosphatase (134.0 vs. 100.81 U/L; *p* = 0.006), CA 19-9 (187.01 vs. 24.32 ng/mL; *p* = 0.001), CEA (9.54 vs. 2.36 ng/mL; *p* = 0.001), AFP (8.03 vs. 4.64 ng/mL; *p* = 0.001), and CA-125 (198.93 vs. 23.59 ng/mL; *p* = 0.001), while amylase values were significantly lower (53.24 vs. 242.08 U/L; *p* = 0.015).

**Figure 3:**
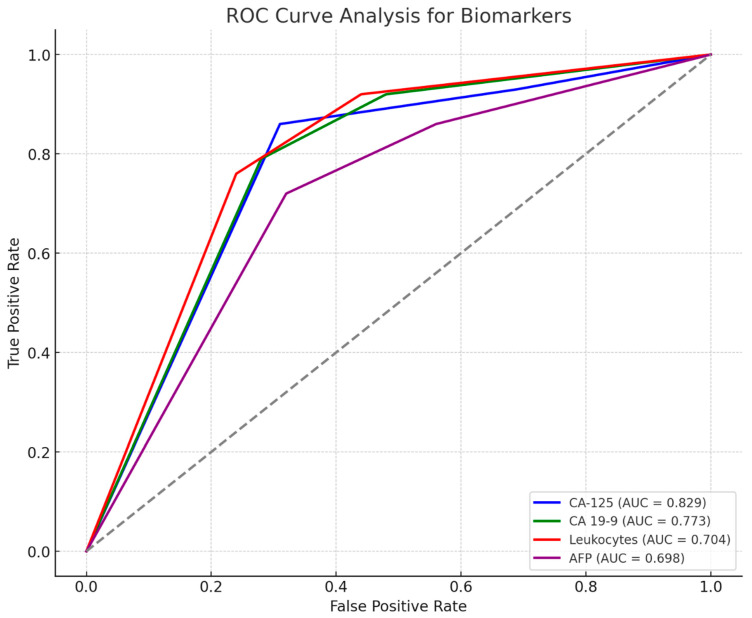
ROC curve. Laboratory parameters predictive of acute cholecystitis.

ROC curve analysis confirmed the following as good predictors of acute cholecystitis: CA-125 (AUC = 0.829; 95% CI: 0.719–0.939; *p* = 0.001), with values greater than 12.30 ng/mL showing a sensitivity of 86% and specificity of 69%; CA 19-9 (AUC = 0.773; 95% CI: 0.654–0.892; *p* = 0.001), with values greater than 11.40 ng/mL showing a sensitivity of 79% and specificity of 72%; leukocytes (AUC = 0.704; 95% CI: 0.591–0.817), with values greater than 8205 n/µL showing a sensitivity of 76% and specificity of 62%; and AFP (AUC = 0.698; 95% CI: 0.577–0.819; *p* = 0.003), with values greater than 3.98 ng/mL showing a sensitivity of 72% and specificity of 52% (see Figure 3).

### 2.5. Analysis of Thrombocyte Counts and Clotting Parameters 

This study evaluated thrombocyte counts and clotting parameters (INR, Prothrombin Activity, aPTT) in relation to tumour markers (CEA, CA19-9, CA125, AFP) within different histopathological groups (adenocarcinoma, benign lesions, metaplasia).

### 2.6. Thrombocyte Counts

Patients with adenocarcinoma exhibited significantly lower thrombocyte counts compared to those without adenocarcinoma (mean: 198,000/µL vs. 267,000/µL, *p* = 0.012). In the metaplasia group, thrombocyte counts were also lower (mean: 215,000/µL), though the difference was not statistically significant (*p* = 0.078). No significant differences in thrombocyte levels were observed between the patients with benign lesions and the metaplasia groups, suggesting that thrombocyte involvement in tumour progression is specific to adenocarcinoma.

We found a statistically significant correlation between thrombocyte count and CA 19-9 levels in the adenocarcinoma group (Pearson correlation coefficient = 0.56, *p* = 0.02; Spearman correlation coefficient = 0.52, *p* = 0.03). This suggests a potential link between platelet levels and tumour progression in adenocarcinoma patients. In contrast, no significant correlations were found between thrombocyte count and other tumour markers (CEA, AFP, and CA 125) (see Table 4).

In patients with benign histopathological findings, no statistically significant correlations were identified between thrombocyte count and tumour markers. The Pearson correlation coefficients for CEA, CA 19-9, AFP, and CA 125 ranged from 0.11 to 0.23, with *p*-values above 0.30, indicating a lack of meaningful associations. Similarly, in the metaplasia group, no significant correlations were found between thrombocyte count and any of the assessed tumour markers. The Pearson correlation coefficients for CEA, CA 19-9, AFP, and CA 125 remained low (ranging from 0.12 to 0.21), with all *p*-values exceeding 0.35.

### 2.7. Variation in Clotting Time Levels

The adenocarcinoma group had the highest INR levels (mean: 1.136), followed by the metaplasia group (mean: 1.059) and the non-adenocarcinoma group (mean: 1.055). Across the three histopathological groups (benign lesions, metaplasia, and adenocarcinoma), INR showed weak correlations with tumour markers. INR exhibited a negative correlation with CEA, suggesting a potential inverse relationship. Similarly, INR correlations with CA 19-9, CA 125, and AFP were weak across all histopathological categories (see Table 5).

Regarding prothrombin activity, the metaplasia group exhibited the highest prothrombin activity (mean: 82.91%), followed by those with adenocarcinoma (mean: 80.72%) and benign lesions (mean: 79.57%). A statistically significant correlation was observed in the group with benign lesions between prothrombin activity and CRP (Spearman correlation = 0.421, *p* = 0.0106). In the metaplasia group, strong but opposite correlations were found between prothrombin activity and CEA and CA19-9.

Concerning aPTT (activated partial thromboplastin time), the adenocarcinoma group had the highest aPTT levels, while the metaplasia group had the lowest aPTT levels. A statistically significant correlation was found between aPTT and CA19-9 in the group with benign lesions (Spearman correlation = 0.369, *p* = 0.02), suggesting that prolonged clotting time may be associated with higher CA19-9 levels in these patients. In the metaplasia group, strong correlations were observed between aPTT and CEA (*p* = 0.001) and CA19-9 (*p* = 0.001) (see Table 6).

### 2.8. Inflammatory Infiltration in Gallbladder Lesions

From the histopathological examinations, 63.3% of the results showed inflammatory infiltration, of which 47.4% cases were observed in patients with chronic cholecystitis, and 29.8% in patients with acute cholecystitis. Regarding the histopathological findings, 41.1% of the results indicated parietal fibrosis, with 37.8% of cases found in patients with acute cholecystitis (*p* = 0.162) and 51.4% in patients with chronic cholecystitis (*p* = 0.001). Parietal fibrosis refers to the formation of fibrous tissue (scarring) in the walls of the gallbladder.

In patients with adenocarcinoma, the level of C-reactive protein (CRP) was significantly influenced by a combination of clinical and laboratory parameters. The analysis revealed that 53% of the variation in CRP levels could be explained by independent variables such as age, hemoglobin (Hb), hematocrit (HT), amylase, CA19-9, and carcinoembryonic antigen (CEA), with a statistically significant model fit (adjusted R^2^ = 0.530, *p* = 0.004).

The stepwise inclusion of these variables in the regression model demonstrated how each factor contributed to refining the predictive value of CRP levels. Initially, age alone showed a weak correlation with CRP. The addition of hemoglobin and hematocrit slightly improved the explanatory power, suggesting that anemia and hematological status may play a minor role in influencing CRP levels. However, the model gained greater strength with the inclusion of amylase, a marker often associated with pancreatic or biliary pathology, indicating a potential link between systemic inflammation and enzymatic activity in these patients.

**Figure 4:**
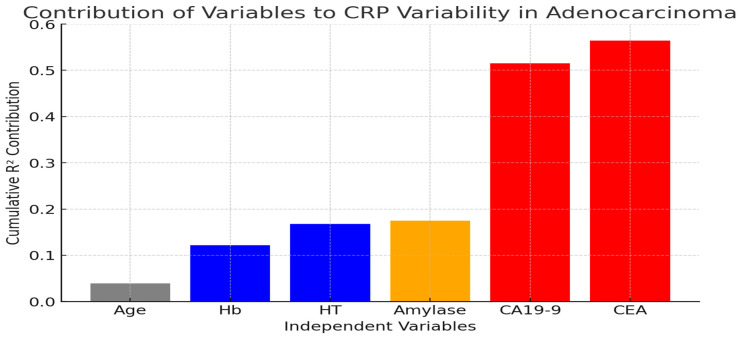
Contribution of independent variables to CRP variability in adenocarcinoma—the figure shows how each independent variable contributes to explaining CRP variability in adenocarcinoma. The progression shows how adding age, hematological parameters, amylase, and tumour markers (CA19-9 and CEA) strengthens the model.

A major improvement in the predictive capacity was observed when CA19-9, a well-known tumour marker for gastrointestinal malignancies, was introduced. The strong association between CA19-9 and CRP suggests that inflammation and tumour burden are closely intertwined in adenocarcinoma. The final key factor, CEA, further enhanced the model, reinforcing the role of tumour markers in systemic inflammatory response regulation. This highlights the intricate interplay between cancer progression and inflammation, where elevated CRP may reflect not only an immune response, but also tumour-related factors that drive disease severity—Figure 4.

Further attempts to refine the model by incorporating additional tumour markers, such as alpha-fetoprotein (AFP) and CA125, did not yield significant improvements in predictive power.

In patients with metaplasia, more than 50% of the CRP value could be explained by the presence of independent variables, including age, hemoglobin (Hb), hematocrit (HT), amylase, CA19-9, and carcinoembryonic antigen (CEA) (adjusted R^2^ = 0.501, *p* = 0.001). This result highlights a significant association between CRP levels and a combination of hematological, enzymatic, and tumour-related markers, reinforcing the role of systemic inflammation in the presence of metaplastic changes.

A stepwise regression analysis was performed to evaluate the impact of each predictor on CRP variability. Initially, age alone accounted for only a small proportion of the variance (adjusted R^2^ = 0.018, *p* = 0.116). The inclusion of Hb and HT progressively improved the model’s predictive power, indicating that hematological status may influence CRP levels in patients with metaplasia. However, the most substantial increase in explanatory power was observed when CA19-9 and CEA were added to the model, with the final combination of these variables leading to the strongest association with CRP levels (adjusted R^2^ = 0.501, *p* = 0.001).

Attempts to further refine the model by adding additional tumour markers, such as alpha-fetoprotein (AFP) and CA125, did not significantly improve its predictive capability, as seen in the negligible increase in R^2^ values and non-significant *p*-values (*p* = 0.480 and *p* = 0.479, respectively).

In patients with chronic cholecystitis, 53% of CRP variability was explained by the presence of independent variables, including age, hemoglobin (Hb), hematocrit (HT), amylase, CA19-9, and CEA (adjusted R^2^ = 0.530; *p* = 0.004). Initially, age alone accounted for only 3.9% of the variance, with an adjusted R^2^ of 0.027 and *p* = 0.074. When hemoglobin was added as an independent variable, the explanatory power increased to 12.2% (adjusted R^2^ = 0.100; *p* = 0.007). The inclusion of hematocrit further improved the model, capturing 16.8% of the variance (adjusted R^2^ = 0.137; *p* = 0.039). However, adding amylase did not significantly enhance the model (adjusted R^2^ = 0.133; *p* = 0.408). The most notable improvement occurred when CA19-9 was introduced, explaining 51.5% of the variance (adjusted R^2^ = 0.484; *p* = 0.001). Finally, the addition of CEA resulted in the best model, accounting for 56.4% of CRP variability with statistical significance (adjusted R^2^ = 0.530; *p* = 0.004)—Table 7.

In patients with parietal fibrosis found at the histopathological exam, over 85% of CRP variability was explained by the same set of independent variables (adjusted R^2^ = 0.853; *p* = 0.031). The model’s explanatory power increased progressively, starting from 7.6% with age alone (adjusted R^2^ = 0.049; *p* = 0.100), and reaching 61.3% with the addition of hematocrit (adjusted R^2^ = 0.578; *p* < 0.001). The most substantial improvement was observed when CA19-9 was included, raising the explained variance to 85.6% (adjusted R^2^ = 0.833; *p* < 0.001). Adding CEA further strengthened the model, allowing it to reach the highest adjusted R^2^ of 0.853 (*p* = 0.031), confirming the strong association between these tumour markers and CRP levels in patients with parietal fibrosis.

For patients with inflammatory infiltration found at the histopathological exam, 58.8% of CRP variability was explained by age, hemoglobin, hematocrit, amylase, and CA19-9 (adjusted R^2^ = 0.588; *p* = 0.001). Age alone contributed to only 7.9% of the variance (adjusted R^2^ = 0.062; *p* = 0.035), with a gradual increase as more variables were included. When hematocrit was added, the model explained 25.5% of the variance (adjusted R^2^ = 0.212; *p* = 0.197). The most significant jump occurred when CA19-9 was introduced, raising the explained variance to 62.5% (adjusted R^2^ = 0.588; *p* = 0.001). However, adding CEA did not lead to further improvement (adjusted R^2^ = 0.590; *p* = 0.262), indicating that CA19-9 was the primary independent variable influencing CRP levels in this group.

## 3. Discussion

This study provides significant insights into the histopathological characteristics, thrombocyte counts, clotting parameters, and tumour marker correlations in patients with gallbladder disease, including adenocarcinoma and metaplasia. The findings highlight key hematological and biochemical variations associated with different gallbladder pathologies, shedding light on potential diagnostic and prognostic markers.

The study demonstrates the significant role of both clinical and laboratory parameters in explaining thrombotic balance variability and inflammatory status, underscoring the importance of these markers in the pathogenic processes associated with gallbladder diseases.

This study cohort was predominantly composed of female patients, a finding that is consistent with prior studies showing a higher prevalence of gallbladder diseases, particularly chronic cholecystitis and gallbladder neoplasia, among women. A significant proportion of patients were elderly, over the age of 65. These findings align with established trends in the epidemiology of gallbladder diseases, where age and gender are crucial factors in determining disease prevalence.

Chronic cholecystitis emerged as the most common diagnosis among the study participants, followed by acute cholecystitis. Both conditions showed a strong association with obesity and hypertension, which have been widely recognized as risk factors for gallbladder disease. These findings reinforce the significance of managing these risk factors to prevent or mitigate the severity of gallbladder-related pathologies.

Among the patients diagnosed with adenocarcinoma, all were female and over the age of 65. This group demonstrated significantly elevated levels of tumour markers such as CA 19-9, CEA, AFP, and CA-125 compared to other patients. The ROC curve analysis revealed that CA 19-9, CEA, AFP, and CA-125 were excellent predictors of adenocarcinoma, with high sensitivity and specificity, especially when their levels exceeded certain thresholds. This reinforces the utility of these markers in diagnosing gallbladder adenocarcinoma, and highlights their potential role in monitoring disease progression and treatment response.

### 3.1. Haemostatic Balance in Gallbladder Lesions

The significantly lower thrombocyte counts in adenocarcinoma patients compared to patients with benign lesions indicate a potential link between thrombocytopenia and gallbladder malignancy. This finding suggests that platelet consumption in tumour microenvironments or cancer-induced hematological alterations may contribute to reduced thrombocyte levels. The trend toward lower thrombocyte counts in metaplasia, though not statistically significant, suggests a possible preneoplastic or inflammatory influence on platelet dynamics.

The literature shows that platelets play a crucial role in the progression and metastasis of neoplasia. The dynamic interaction between platelets and tumour cells promotes tumour growth, angiogenesis, metastasis, and dissemination. Research has consistently shown that modified platelet levels correlate with poor prognosis in various neoplasms, including pancreatic, gastric, colorectal, endometrial, and ovarian neoplasia [12,13]. However, platelet count is regulated by the balance between production and consumption. A normal platelet count may mask a highly hypercoagulable and pro-inflammatory neoplastic phenotype due to effective compensatory mechanisms. Platelets play a crucial role in the physiological process of hemostasis, as well as in tumour growth and metastasis. They have been observed to adhere to tumour cells, aggregate, and locally release angiogenic factors, which are hypothesized to interact with both tumour cells and vascular endothelial cells, contributing to both physiological and pathological angiogenesis [14].

Platelets serve as a major source of platelet-derived endothelial cell growth factor (TP/PD-ECGF), a molecule with mitogenic and pro-angiogenic properties. They can endocytose and store TP/PD-ECGF in their α-granules, rapidly secreting it upon activation. Yamamoto et al. reported that TP/PD-ECGF, which induces endothelial cell chemotaxis in vitro and exhibits angiogenic activity in vivo, is produced by cancer cells and infiltrating cells involved in tumour progression in human gallbladder cancer (GBC) [15]. Additionally, platelets uptake and concentrate vascular endothelial growth factor (VEGF) secreted by tumour cells, later transporting it within their granules. Recent studies suggest that platelet–tumour cell interactions enhance metastasis by promoting epithelial–mesenchymal transition (EMT) through the TGFB/SMAD and NFKB pathways. Notably, inhibiting these pathways specifically in platelets was found to suppress metastasis in vivo. Furthermore, platelets contribute to tumour immune evasion by expressing immunoregulatory proteins, such as glucocorticoid-induced TNF-related protein, which protect tumour cells from the host’s immune response. Intratumoural platelet activation and the subsequent release of thrombopoietin may contribute to increased platelet production. Additionally, the thrombopoietic cytokine interleukin-6, produced by tumour tissues, has been linked to platelet levels. Overall, the interplay between platelets and tumour cells significantly drives tumour progression [16].

Due to the interplay between tumour-induced anticoagulant activity and the host fibrinolytic system, patients with neoplasia may experience significant alterations in coagulation and fibrinolysis-related biomarker levels. Common coagulation and thrombosis indicators include platelet count (PLT), prothrombin activity (PA), INR, activated partial thromboplastin time (APTT), and fibrinogen (FIB). These traditional coagulation markers are routinely used to evaluate bleeding, coagulation, and thrombotic tendencies. However, tumour-related metabolic changes, metastasis, and toxin production can also influence their levels.

Although these markers provide insight into the hemostatic status of patients with neoplasia, they lack specificity for tumour detection [17].

Increased INR or prolonged PT signifies an impaired extrinsic coagulation pathway. Neoplasia-associated activation of the coagulation system can lead to the depletion of coagulation factors, contributing to PT prolongation. Additionally, the reduced biosynthetic capacity of the liver in patients with neoplasia may further exacerbate this condition. Previous studies have demonstrated that prolonged PT is linked to poorer prognoses across various types of neoplasia. Ke et al. showed that an elevated INR was associated with poor tumour differentiation, prolonged prothrombin time and activated partial thromboplastin time, an increased activated partial thromboplastin time ratio, and reduced prothrombin activity in neoplastic patients. These factors were identified as adverse prognostic indicators in univariate analysis. Additionally, a high international normalized ratio correlated with lower albumin levels, a marker of liver dysfunction and poor nutritional status [18].

The relationship between clotting profile and tumour markers across different histopathological groups was explored in this study. Overall, INR demonstrated weak correlations with tumour markers. These findings suggest that INR alone may not be a reliable marker for tumour burden in gallbladder pathology. Notably, a statistically significant negative correlation was found between INR and CEA in the adenocarcinoma group, suggesting that higher INR values may be associated with lower CEA levels in patients with adenocarcinoma. This finding could imply a complex interplay between coagulation status and tumour progression, potentially reflecting an altered hemostatic balance in these patients.

Conversely, INR correlations with other tumour markers, including CA 19-9, CA 125, and AFP, remained weak across all histopathological groups. The absence of significant associations suggests that while coagulation abnormalities are frequently observed in malignancies, INR alone does not appear to be a strong predictor of tumour marker elevation in gallbladder neoplasms.

A statistically significant correlation was identified between prothrombin activity and C-reactive protein in the non-adenocarcinoma group, suggesting a possible link between coagulation function and systemic inflammation in these patients. Given that inflammation plays a crucial role in neoplasia progression and hemostatic disturbances, this finding warrants further investigation into the interplay between inflammatory response and coagulation in non-adenocarcinoma cases.

In the metaplasia group, prothrombin activity demonstrated strong but opposing correlations with CEA and CA 19-9. However, these extreme correlation values may have been influenced by the small sample size, rather than reflecting a true biological association. Similarly, strong correlations were observed in the metaplasia group between APTT and both CEA and CA 19-9.

A notable finding in the non-adenocarcinoma group was the correlation between APTT and CA 19-9, suggesting that prolonged clotting time may be associated with higher tumour marker levels. While previous studies have indicated that hypercoagulability is linked to tumour progression, this result may reflect a more complex interaction between tumour burden, coagulation abnormalities, and inflammatory status in non-adenocarcinoma patients.

Overexpression of procoagulant proteins is a key factor contributing to the abnormal activation of coagulation in patients with malignant neoplasia. Tumour cell-derived tissue factor, also known as Factor III, plays a significant role in neoplasia-associated coagulopathy [10]. By binding with Factor VIIa, tissue factor initiates the coagulation cascade by activating Factor X, making it a crucial component of the extrinsic coagulation pathway. The extrinsic pathway appears to be more disrupted than the intrinsic pathway in neoplastic conditions. Consequently, the international normalized ratio, which reflects the extrinsic pathway based on prothrombin time and prothrombin activity, showed a stronger correlation with the prognosis of biliary tract neoplasia than with intrinsic pathway indicators, such as activated partial thromboplastin time and activated partial thromboplastin time ratio [19].

The fibrinolytic system also contributes to hemostatic imbalances in patients with neoplasia. While urokinase-type plasminogen activator, tissue-type plasminogen activator, and their receptors are commonly activated during tumour progression, fibrinolysis inhibitors are also upregulated. This contradictory regulation may explain why thrombin time, an indicator of the fibrinolytic system, did not emerge as an independent prognostic factor for biliary tract neoplasia. Additionally, platelets are known to be involved in neoplasia-associated coagulopathy and disease progression [20].

Previous research by Wang et al. demonstrated that prolonged prothrombin time was associated with higher recurrence rates and poor prognosis in patients with intrahepatic cholangiocarcinoma following curative resection [21]. Similarly, a retrospective and in vitro study by Shu et al., conducted on a relatively small cohort (*n* = 115), highlighted the clinical significance of elevated preoperative fibrinogen levels in patients with gallbladder neoplasia after surgical resection [22]. Although coagulopathy has been linked to adverse outcomes in intrahepatic cholangiocarcinoma and gallbladder neoplasia, data regarding the prognostic significance of coagulation indices in biliary tract neoplasia remain limited. Furthermore, few prognostic models based on coagulation parameters have been established for biliary tract neoplasia. Therefore, further comprehensive investigations are needed to clarify the prognostic value of coagulation indices and their relationships with other clinical characteristics in this disease.

Although the American Joint Committee on Cancer TNM staging system is widely used to assess clinical outcomes in patients with biliary tract neoplasia, prognosis varies, even among patients classified within the same TNM stage. Other pathological characteristics, such as tumour subtype and degree of differentiation, are also considered in survival assessments. However, it is now widely recognized that prognostic outcomes are influenced not only by tumour-intrinsic factors, but also by patient-related characteristics. Given that coagulation markers, particularly fibrinogen and the international normalized ratio, were found to be associated with overall survival in biliary tract neoplasia, a nomogram incorporating five independent prognostic factors identified through multivariate analysis was developed. This model integrates tumour-related, patient-related, and treatment-related factors, demonstrating strong predictive performance in terms of discrimination, consistency, and clinical benefit.

Malignancy disrupts the hemostatic system, predisposing patients to both thrombosis and hemorrhage. The link between neoplasia and thrombosis was first described by Armand Trousseau in 1865, highlighting a hypercoagulable state or chronic disseminated intravascular coagulation in these patients. Tumour cells contribute to coagulation activation by expressing prothrombotic properties, leading to thrombin and fibrin generation, platelet activation, and endothelial dysfunction, all of which promote neoplastic progression.

Venous thromboembolism (VTE) is the most studied thrombotic complication in neoplasia, with patients facing a sevenfold increased risk compared to the general population. This risk has risen due to improved neoplasia survival, more thrombogenic therapies, and an ageing population. Thrombosis can also precede neoplasia diagnosis by months or years. Various factors influence thrombotic risk, including immobility, advanced disease stage, and neoplasia treatments. While Trousseau initially described venous thrombosis in gastric neoplasia, “Trousseau’s syndrome” now encompasses a range of thromboembolic events in malignancy, including arterial and venous thrombosis, non-bacterial thrombotic endocarditis, thrombotic microangiopathy, and veno-occlusive disease [20,22].

In the venous system, deep vein thrombosis of the lower limbs is the most common thrombotic manifestation, followed by upper-limb deep vein thrombosis, pulmonary embolism, cerebral sinus thrombosis, and migratory superficial thrombophlebitis. Large-scale studies estimate the venous thromboembolism (VTE) incidence as being between 0.6% and 7.8%, with neoplasia type being a major determinant of risk. Arterial thromboembolic events (ATEs) in neoplasia are less studied, but include ischemic stroke, myocardial infarction, and peripheral arterial occlusion. The estimated ATE incidence in neoplastic patients ranges from 2% to 5%, accounting for 10–30% of all thrombotic events. Among patients receiving chemotherapy, the symptomatic ATE incidence has been reported at 0.27%. Additionally, patients may experience microcirculatory disturbances, leading to transient ischemic attacks, visual and auditory impairments, headaches, and paresthesia. Non-bacterial thrombotic endocarditis (NBTE) is particularly common in myeloproliferative neoplasms, but also occurs in solid neoplasms, affecting 0.9–1.3% of neoplastic patients at autopsy. This condition results from systemic coagulation activation, leading to platelet and fibrin vegetations on cardiac valves. These vegetations can embolize, causing strokes, splenic infarctions, or acute limb ischemia. Other thrombotic complications include thrombotic microangiopathy (TMA) and veno-occlusive disease (VOD) [23]. TMA is a rare but severe condition in neoplasia, characterized by microangiopathic hemolytic anemia, thrombocytopenia, and organ dysfunction. It can manifest as thrombotic thrombocytopenic purpura or hemolytic uremic syndrome, and has been associated with chemotherapy agents such as mitomycin, gemcitabine, and targeted therapies like monoclonal antibodies and tyrosine kinase inhibitors. Thrombosis can also be an early sign of undiagnosed neoplasia. Patients with idiopathic VTE have a four- to sevenfold increased risk of neoplasia diagnosis within a year, with an even higher risk in patients with recurrent or bilateral VTE. However, the need for extensive neoplasia screening in patients with unprovoked VTE remains debated, as studies comparing limited and extensive screening have yielded inconclusive results.

At the opposite end of the spectrum, hemorrhagic complications are a significant cause of mortality in neoplasia, affecting around 10% of patients with solid neoplasms and a higher percentage of those with hematologic neoplasms. Bleeding may present as gastrointestinal, urinary, respiratory, or gynecological hemorrhages, as well as skin lesions, bruising, and petechiae. It can occur acutely or as recurrent low-grade bleeding due to thrombocytopenia, coagulation factor deficiencies, vitamin K deficiency, anticoagulant therapy, or disseminated intravascular coagulation (DIC). Extremely high platelet counts can lead to acquired von Willebrand disease, increasing bleeding risk, a mechanism also observed in lymphoproliferative disorders [24,25].

DIC is characterized by widespread intravascular coagulation activation, leading to both thrombosis and bleeding. In this state, fibrinolysis is also activated, resulting in excessive consumption of clotting factors and platelets. D-dimer measurement helps to assess fibrinolysis levels in DIC. Data on DIC in solid neoplasms remain limited, with one study reporting a 7% incidence.

Another rare but severe bleeding disorder in neoplasia is acquired hemophilia, caused by autoantibodies against coagulation factors, most commonly factor VIII. Given its potential for life-threatening hemorrhages, acquired hemophilia should be considered in neoplastic patients presenting with unexplained bleeding [26].

### 3.2. Tumour Marker Assessment

The association between elevated tumour markers and CRP levels in adenocarcinoma patients suggests a close relationship between inflammation and cancer progression. The CRP levels in these patients may not only reflect an inflammatory response, but also indicate the presence of malignancy. The inclusion of tumour markers such as CA 19-9 and CEA in the regression model improved the explanatory power, underscoring the role of neoplasia-related inflammation in the systemic response observed in adenocarcinoma.

Our findings regarding metaplasia in gallbladder lesions are also significant. Metaplasia was most commonly associated with chronic cholecystitis. The results of this study indicate that obesity and hypertension are major risk factors for the development of metaplasia, with a particularly high relative risk in obese and hypertensive patients. Additionally, patients with metaplasia exhibited significantly elevated amylase and creatinine levels, as well as lower hemoglobin levels, suggesting a complex interaction between metabolic, enzymatic, and hematological factors in the disease process.

The ROC curve analysis confirmed that amylase, CEA, CA 19-9, and creatinine were strong predictors of metaplasia. The high sensitivity and specificity values for these markers demonstrate their potential in identifying patients at risk for metaplastic changes, which may serve as precursors to more severe gallbladder pathologies, including adenocarcinoma.

Chronic cholecystitis was associated with obesity, hypertension, and elevated levels of liver enzymes, particularly AST and ALT. These findings align with the existing literature that highlights the role of metabolic and inflammatory factors in the development of chronic cholecystitis. The stepwise regression analysis demonstrated that the combination of clinical and laboratory parameters, including AST, ALT, amylase, and tumour markers like CA 19-9, provided a strong predictive model for CRP levels in patients with chronic cholecystitis. This suggests that systemic inflammation and liver function are intertwined in the pathophysiology of chronic cholecystitis.

Acute cholecystitis showed a strong association with elevated levels of leukocytes, alkaline phosphatase, CA 19-9, CEA, AFP, and CA-125. The significant differences in these markers between patients with acute cholecystitis and those with other gallbladder conditions underscore the inflammatory nature of this condition. The stepwise regression model highlighted CA 19-9 and CA-125 as strong predictors of acute cholecystitis, with high sensitivity and specificity, which could aid in early diagnosis and differentiation from other forms of gallbladder disease.

In patients with parietal fibrosis and inflammatory infiltration, the combination of age, hemoglobin, hematocrit, amylase, and CA 19-9 was able to explain a large proportion of the variability in CRP levels. These findings suggest that systemic inflammation, as evidenced by elevated CRP, is closely linked with fibrosis and inflammatory changes in the gallbladder. The presence of these pathological features may reflect a chronic inflammatory state that predisposes individuals to further complications, including cancer.

Carcinoembryonic antigen (CEA) and carbohydrate antigen 19-9 (CA19-9) are widely recognized cancer-associated markers that are commonly used to aid in the diagnosis and prognosis of digestive system cancers. These markers have shown to be particularly valuable in assessing pancreatic, colorectal, stomach, and bile duct cancers [12]. Preoperative CA19-9 levels were shown to predict tumour resectability in a study of 292 patients with stage I-IV gallbladder carcinoma. In early-stage cases (0-I), radical resection is more common and associated with favourable outcomes, with over 60% of patients achieving five-year survival. These findings highlight the importance of tumour markers like CEA and CA19-9 not only for diagnosis, but also for assessing surgical success and prognosis [27,28,29,30,31].

### 3.3. Carbohydrate Antigen 19-9 (CA 19-9)

Carbohydrate antigen 19-9 (CA 19-9) is an important tumour marker used for the detection and monitoring of various cancers, particularly those affecting the digestive system. Initially identified in patients with colorectal cancer, CA 19-9 is now most employed to diagnose pancreatic, bile duct, and gallbladder cancers [32]. Although elevated CA 19-9 levels are not exclusive to any neoplasia, they are often associated with gastrointestinal cancers, including gallbladder and pancreatic cancers. The specificity of CA 19-9 can be influenced by non-cancerous conditions such as pancreatitis, liver diseases, or bile duct obstruction. Despite these limitations, CA 19-9 remains useful in assessing tumour burden, predicting the likelihood of tumour resectability, and monitoring disease progression or recurrence after treatment. Given that its sensitivity and specificity can vary depending on the type and stage of the neoplasm, CA 19-9 is frequently used in combination with other tumour markers to improve diagnostic accuracy [33,34,35]. Sachan et al. investigated the prognostic value of CA19-9 and CEA in gallbladder cancer, concluding that CA19-9 was superior in assessing tumour burden and predicting recurrence [36]. Similarly, Bind et al. evaluated CA19-9 and CA125 levels for diagnosis and prognosis in gallbladder cancer, finding CA19-9 to be a more reliable prognostic marker than CA125. Moreover, Wang et al. reported that CA 19-9 showed the highest sensitivity and specificity, with values of 71.7% and 98.7%, respectively [16]. Serum CA 19-9, in particular, demonstrated strong specificity and moderate sensitivity, indicating its potential role in detecting GBC. However, when used alone, these tumour markers have shown varying results, with differing sensitivity and specificity across various studies. Importantly, combining these markers improves the sensitivity of GBC diagnosis.

When both CA 19-9 and CEA levels surpassed their critical thresholds, the sensitivity was 3.8%. This finding is consistent with another study that reported an 8.9% increase in diagnostic accuracy and sensitivity when three markers—CA 19-9, CA 125, and CA 242—were used together. These findings suggest that the use of multiple tumour markers can significantly enhance the specificity and sensitivity of GBC diagnostics, providing a more dependable method for early disease detection and accurate assessment [10,15,37].

### 3.4. Carcinoembryonic Antigen (CEA)

Carcinoembryonic antigen (CEA) is a widely studied tumour marker in various malignancies, including gallbladder cancer. While its primary role has been in colorectal cancer monitoring, research has shown that elevated CEA levels may be associated with tumour burden, disease progression, and prognosis in gallbladder cancer. Studies suggest that CEA, in combination with other markers such as CA19-9, can enhance diagnostic accuracy and provide prognostic insights, particularly in advanced stages of the disease. However, its specificity for gallbladder cancer remains limited, as elevated levels can also be observed in benign conditions such as cholecystitis and other gastrointestinal malignancies. However, its specificity for GBC is limited due to the possibility of increased levels in non-cancerous conditions, such as inflammation or biliary obstruction [38]. Despite these limitations, CEA remains valuable for monitoring tumour burden and treatment response and detecting potential recurrence after surgery when used alongside other markers, like CA 19-9. This combination improves diagnostic accuracy, especially since GBC often presents at advanced stages, where early detection using combined tumour markers can enhance treatment outcomes [39].

CA19-9 and CEA are two of the most commonly used tumour markers for gallbladder neoplasia (GBC). Further research is needed to determine their role in predicting metastasis or unresectability. Accurate assessment of serum levels of these markers can enhance the accuracy of prognostic evaluations for patients. In contrast to CEA, Wang et al. [10] found that CA19-9 levels increased progressively with advancing clinical stages. Current data show that, unlike CEA levels, which do not significantly correlate with tumour formation, CA19-9 levels rise with increasing tumour burden. A study by Shukla PJ et al., involving 335 patients, 80 of whom had jaundice, showed that in 95% of cases, CA19-9 levels greater than 90 IU/mL were associated with incurable disease [40].

Additionally, the 5-year survival rate for patients with CEA levels ≤4 ng/mL was 42.8%, while for those with levels >4 ng/mL, it was only 12.5%. Similarly, the 5-year survival rate for patients with CA19-9 levels ≤37 U/mL was 40%, whereas no patients with CA19-9 levels >37 U/mL survived beyond 5 years, even after successful surgical resection. These findings suggest that preoperative levels of CEA and CA19-9 may help to assess a patient’s prognosis before curative surgery. In a study by Agarwal and colleagues, GBC patients who underwent extended cholecystectomy and had CA19-9 levels below 20 IU/mL had a 4-year survival rate of 78%, while those with levels above 20 IU/mL had a 3-year survival rate. Patients with CA19-9 levels <20 IU/mL did not reach median survival, while those with levels >20 IU/mL had a 12-month median survival [39,40,41].

The histopathological analysis of the patients included in our study revealed that 6.7% of cases had adenocarcinoma, exclusively affecting female patients over the age of 65 who also presented with acute cholecystitis. These findings suggest a strong association between gallbladder malignancy and advanced age, female sex, and inflammatory conditions such as acute cholecystitis. Laboratory investigations demonstrated that patients with adenocarcinoma had significantly elevated levels of CA 19-9, CEA, AFP, and CA-125, while amylase and total bilirubin levels were notably lower. These biomarker trends underscore their potential role in aiding the early detection of adenocarcinoma in at-risk patients.

The ROC curve analysis validated several laboratory parameters as reliable predictors of adenocarcinoma. CEA exhibited the highest predictive power (AUC = 1.000), with a sensitivity and specificity of 100% at values exceeding 25 ng/mL. CA-125 and CA 19-9 also demonstrated strong predictive capabilities, with high sensitivity and specificity at cutoff values of 319 ng/mL and 307 ng/mL, respectively. Similarly, AFP proved to be a reliable marker, achieving 100% sensitivity and 97% specificity at values above 12.55 ng/mL. These findings highlight the diagnostic utility of these biomarkers, particularly when used in combination, for improving the early identification of gallbladder adenocarcinoma.

Regarding metaplasia, 7.8% of cases were histopathologically classified as such, again exclusively in female patients, with the majority being over 65 years of age and all presenting with chronic cholecystitis. These observations reinforce the notion that chronic gallbladder inflammation plays a significant role in the development of metaplastic changes, which may serve as a precursor to malignancy. The estimated risk of metaplasia was significantly elevated in obese patients (RR = 12.96) and those with hypertension (RR = 14.89), suggesting that metabolic and cardiovascular comorbidities contribute to gallbladder epithelial alterations.

The ROC curve analysis further identified key laboratory markers predictive of metaplasia. Amylase exhibited an AUC of 0.873, with a sensitivity of 100% and a specificity of 71% at values above 59 U/L. CEA and CA 19-9 were also strong predictors, with high sensitivity and specificity at respective cutoff values of 1.46 ng/mL and 13.57 ng/mL. Additionally, creatinine emerged as a reliable predictor (AUC = 0.880), with a sensitivity of 100% and specificity of 78% at values exceeding 1.01 mg/dL. These findings indicate that biochemical markers can be instrumental in identifying patients at higher risk for metaplastic transformation and, potentially, subsequent malignancy.

The use of CA 19-9 and CEA together has shown significant potential for predicting adenocarcinoma and metaplasia in our study. In cases of gallbladder adenocarcinoma, we observed that elevated levels of both CA 19-9 and CEA were strong indicators of malignancy, with a prediction score of 2 (both markers above their respective cutoffs) being strongly associated with the presence of adenocarcinoma. Specifically, when CA 19-9 levels exceeded 307 ng/mL and CEA levels exceeded 25 ng/mL, the likelihood of adenocarcinoma was notably higher. This combined scoring system enhances early detection and prognosis by utilizing both biomarkers, which, when assessed individually, already exhibit high sensitivity and specificity. Likewise, in the cases of metaplasia observed in our study, a combined score of 2 (both CA 19-9 levels exceeding 13.57 ng/mL and CEA levels exceeding 1.46 ng/mL) corresponded with an increased likelihood of metaplastic changes, highlighting the potential for progression towards malignancy. When both biomarkers are elevated, this dual-marker approach significantly improves diagnostic accuracy. On the other hand, when both CA 19-9 and CEA levels are below their respective thresholds, the prediction score of 0 suggests a reduced risk of either adenocarcinoma or metaplasia. Our findings demonstrate that the combination of CA 19-9 and CEA offers a more reliable method for early identification and risk assessment of gallbladder malignancies and metaplasia. This approach, especially when considering clinical factors such as advanced age, obesity, and hypertension, may greatly enhance the diagnostic and prognostic evaluation of at-risk patients in clinical practice.

Our study underscores the significance of integrating histopathological findings with laboratory biomarkers to enhance the early detection of gallbladder adenocarcinoma and metaplasia. The identification of specific risk factors, such as advanced age, female sex, chronic cholecystitis, obesity, and hypertension, further informs the need for targeted screening and intervention strategies. Future research should explore the molecular mechanisms underlying these associations, and assess the utility of combining these biomarkers in a predictive diagnostic model to improve clinical outcomes.

Tumour markers such as CEA, CA 19-9, AFP, and CA-125 play a crucial role in the early detection and prognostic evaluation of gallbladder lesions. While these markers individually provide varying degrees of sensitivity and specificity, their combined use significantly enhances diagnostic accuracy. Elevated levels of CA 19-9 and CEA have been particularly associated with gallbladder malignancies, whereas CA-125 and AFP contribute additional predictive value. Despite their potential, tumour markers should be interpreted alongside imaging and histopathological findings to improve diagnostic confidence and guide clinical decision-making.

### 3.5. Inflammation in Gallbladder Lesions

Gallbladder inflammation plays a significant role in neoplasia development, but it is not solely driven by gallstones. While gallstones are a common cause, other factors contribute to chronic inflammation, including acalculous cholecystitis, chronic typhoid infection, and xanthogranulomatous cholecystitis. The occurrence of incidental gallbladder neoplasia (IGN), diagnosed unexpectedly without prior suspicion of malignancy, further highlights this connection, with reported incidence rates ranging from 0.19% to 3.3% [20]. These various inflammatory conditions, regardless of gallstone presence, are crucial in understanding the pathogenesis of gallbladder neoplasia [42].

Therefore, we can say that gallbladder neoplasia is an example of chronic inflammation-associated malignancies, developing within a microenvironment shaped by infectious, chemical, mechanical, metabolic, and hydrodynamic stresses over decades. Chronic inflammation drives a cascade of molecular alterations, fueling tumour initiation, adaptation, and progression. Beyond the broader inflammatory setting, the tumour microenvironment itself is highly complex, consisting of diverse cellular populations that interact to sustain tumour growth and immune evasion. Recent single-cell RNA sequencing studies have provided novel insights into these interactions, revealing a dynamic interplay between immune suppression, metabolic reprogramming, and extracellular matrix remodelling.

A key finding in gallbladder neoplasia is the transformation of chronic inflammation into an immunosuppressive tumour microenvironment, facilitated by regulatory T-cells, myeloid-derived suppressor cells, and immune-modulating fibroblasts. This environment not only promotes tumour progression, but also limits the effectiveness of immune surveillance. Additionally, two distinct immunosuppressive subtypes have been identified—one heavily infiltrated with suppressive immune cells, and another characterized by alternative tumour-supportive niches, such as TREM2+ macrophages and tumour-associated fibroblasts. These differences suggest that inflammation-driven neoplasia is not uniform, reinforcing the need for tailored therapeutic approaches.

The metastatic process in gallbladder neoplasia appears to follow a collective migration model, with minimal genomic alterations, but significant translational reprogramming that enhances cancer cell plasticity. The metastatic microenvironment is further shaped by senescence-like fibroblasts, which secrete inflammatory and growth factors that drive angiogenesis, epithelial–mesenchymal transition, and niche formation. Immune evasion mechanisms also evolve, with reduced T-cell cytotoxicity, increased regulatory T-cell activity, and a paradoxical role of interferon signalling in both immune resistance and metastasis [43].

These findings have important therapeutic implications. The metaplastic and inflammatory classifications of gallbladder neoplasia may inform personalized chemotherapy strategies, similar to subtype-driven treatment in ampullary carcinoma. Targeting senescence-like fibroblasts in the tumour stroma also presents a promising avenue for inhibiting metastasis. Understanding these molecular and cellular dynamics will be critical in advancing precision medicine for gallbladder neoplasia.

### 3.6. The Importance of Predictive Scoring Models

In their study, Utsumi et al. investigated the prognostic value of the Prognostic Nutritional Index (PNI) in patients undergoing surgical resection for gallbladder cancer (GBC) [44]. They found that a low PNI was significantly associated with poor postoperative outcomes, aligning with previous research suggesting its role as an independent prognostic factor. In addition, tumour-related features such as lymph node metastasis and poor tumour differentiation were also identified as independent predictors of survival in their multivariate analysis. Based on these findings, they developed a novel inflammation-based prognostic scoring system that combined the PNI with key pathological characteristics. This composite score demonstrated superior prognostic accuracy compared to the use of the PNI or pathological factors alone, suggesting its potential utility in refining risk stratification for GBC patients. Their model was grounded in the understanding that the PNI—calculated from serum albumin levels and total lymphocyte count—reflects both the nutritional and immunological status of cancer patients. A lower PNI indicated a state of systemic inflammation, immunosuppression, and malnutrition, all of which are linked to accelerated tumour progression and reduced survival. In the context of gallbladder cancer, this weakened physiological state likely contributed to worse outcomes following surgery. Furthermore, they observed that patients with a low PNI were more likely to present with advanced disease features, such as lymph node metastasis and longer operation times. These associations further supported the value of the PNI as a marker of aggressive tumour biology [44].

In their study, Kim et al. developed a predictive scoring model to differentiate adenomatous from cholesterol gallbladder polyps (GBPs), addressing the limitations of current size-based diagnostic criteria. They identified three independent predictors for adenomatous polyps: low bile viscosity (<7.5 s^−1^, OR = 22.539), low bile cholesterol levels (<414.5 mg/dL, OR = 10.004), and age over 55 years (OR = 23.550) [45]. When combined into a scoring system, these factors achieved high diagnostic performance, with a sensitivity of 90.9%, specificity of 80.2%, and an AUC of 0.845. This performance surpassed that of previous imaging-based methods, including contrast-enhanced ultrasound and size-based assessments. For instance, contrast-enhanced ultrasound showed a sensitivity and specificity of 76.5% and 75% [46], while the 1 cm threshold for surgical indication yielded only 68.1% and 70.2% [47]. Kim et al.’s model offers a biochemical and clinical approach that more accurately reflects the pathophysiological differences between polyp types. The lower bile viscosity observed in adenomas, potentially linked to reduced mucin secretion, contrasts with cholesterol polyps, where increased mucin expression (e.g., MUC3, MUC5B) raises bile viscosity and contributes to benign pathology. In addition to biochemical markers, age emerged as a significant factor, with a cutoff of 55 years—earlier than commonly cited thresholds—demonstrating strong predictive value. This highlights the potential for earlier identification and intervention in patients at risk of malignancy. Importantly, adenomatous polyps possess malignant potential through an adenoma–carcinoma sequence, reinforcing the urgency of distinguishing them from benign cholesterol polyps. Accurate classification can help to optimize surgical decision-making, ensuring timely cholecystectomy for high-risk lesions, while avoiding unnecessary surgery in benign cases, reducing patient risk and healthcare costs.

Our study presents a novel predictive scoring model focused specifically on gallbladder malignancies and metaplasia, offering a distinct approach from existing models by integrating clinical, biochemical, and histopathological data. Unlike prior models that rely heavily on radiologic size criteria or isolated lab values, our approach combines elevated levels of CA 19-9 and CEA with clinical factors such as advanced age, obesity, and hypertension. This multi-parameter method provides a more comprehensive and reliable stratification of malignancy risk in gallbladder pathology.

Our findings demonstrate that the combination of CA 19-9 and CEA offers a more reliable method for early identification and risk assessment of gallbladder malignancies and metaplasia. This approach, especially when considering clinical factors such as advanced age, obesity, and hypertension, may greatly enhance the diagnostic and prognostic evaluation of at-risk patients in clinical practice. Clotting parameters and inflammation emerged as critical components in understanding the pathophysiology of gallbladder disease. Prothrombin activity showed a significant correlation with C-reactive protein (CRP) in non-adenocarcinoma patients, highlighting a possible link between coagulation and systemic inflammation. This relationship supports the role of inflammatory dysregulation in disease progression, and warrants further exploration. In the metaplasia group, notable associations were observed between coagulation markers (prothrombin activity and APTT) and tumour markers (CEA and CA 19-9), though the small sample size limits definitive conclusions. Still, these findings suggest a potential interplay between clotting abnormalities and early neoplastic changes. Similarly, APTT correlated with CA 19-9 in non-adenocarcinoma cases, implying that prolonged clotting time may be a marker of heightened inflammatory and tumour activity. These insights emphasize the value of incorporating both inflammatory and hemostatic biomarkers into predictive models. Such integration may improve early detection and prognostic accuracy in gallbladder neoplasia and related pathologies.

## 4. Materials and Methods

### 4.1. Design and Ethical Approval

We conducted an observational study between October 2019 and August 2023 at the General Surgery Clinic and the Department of Biochemistry, affiliated with the University of Medicine and Pharmacy, Iasi. The study included 90 patients and received approval from the institutional ethics committees of both the hospital and university, adhering to the ethical principles outlined in the Helsinki Declaration.

### 4.2. Patient Selection and Criteria

Eligible participants were adults (≥18 years) with a high suspicion of gallbladder lesions based on radiological findings, including irregular gallbladder wall thickening, the presence of a mass lesion, polyps, or a porcelain gallbladder. Patients with metastatic GBC, those undergoing chemotherapy or radiation therapy, and individuals diagnosed with a concurrent second primary malignancy were excluded from the study.

### 4.3. Data Collection and Laboratory Assessment

Fasting venous blood samples were collected from all participants preoperatively. Recorded patient data included demographic characteristics (age, weight, and body mass index [BMI]) and biochemical parameters. Laboratory evaluations comprised complete blood counts, liver and kidney function tests, clotting profile parameters, inflammatory markers, and tumour markers. The clotting profile consisted of thrombocyte count, INR, prothrombin activity, and APTT.

To assess the role of inflammation in gallbladder lesions, C-reactive protein (CRP) levels were measured using an immunoturbidimetric assay, with a reference range of <5 mg/L. Tumour markers, including CEA, CA 19-9, AFP, and CA-125, were analyzed using electrochemiluminescence immunoassay. Cutoff values for biomarkers were determined using Youden’s Index from ROC analysis, which identifies the threshold that maximizes the sum of sensitivity and specificity. This method was applied consistently across all variables included in the scoring model.

### 4.4. Histopathological and Clinical Classification

Following surgical intervention, histopathological examination (HPE) was performed on all specimens. Patients were classified into the benign gallbladder disease (GBD) group or the GBC group based on HPE findings. The presence of metaplasia was noted in cases with chronic inflammatory changes. Cancer staging was determined using the TNM classification system (8th edition, 2017) established by the American Joint Committee on Cancer. Inflammatory infiltration refers to the presence of immune cells in the tissue, a common sign of inflammation.

## 5. Limitations and Future Directions

This study offers valuable insights into hemostasis, inflammation, and tumour markers in patients with gallbladder diseases. However, several limitations should be acknowledged. The observational, cross-sectional design restricts the ability to establish causal relationships. Additionally, the relatively small sample size and the predominance of female patients may limit the generalizability of the findings, particularly for males. Future research with larger, more diverse cohorts is necessary to validate these results and further investigate the mechanisms of inflammation in gallbladder pathologies.

While a key limitation of this study is the small sample size, particularly in the subgroups with adenocarcinoma and metaplasia—which may limit the generalizability and statistical strength of the subgroup analyses—the value of our findings lies in their focus on gallbladder malignant lesions. These lesions, although rare, are associated with a dismal prognosis due to delayed detection. Furthermore, although the ROC analyses demonstrated promising diagnostic performance, the overlap in some confidence intervals warrants cautious interpretation and highlights the need for validation in larger, prospective cohorts.

Moreover, while tumour markers such as CA 19-9, CEA, AFP, and CA-125 emerged as strong predictors of adenocarcinoma and metaplasia, further studies are needed to assess their relevance in other gallbladder diseases and confirm their predictive value in clinical practice.

## 6. Conclusions

This study highlights the crucial role of hemostatic imbalance and systemic inflammation in the pathogenesis and prognosis of gallbladder diseases, particularly adenocarcinoma, metaplasia, and chronic cholecystitis. The strong predictive value of tumour markers such as CA 19-9 and CEA underscores their potential as diagnostic and prognostic tools in gallbladder pathologies. These findings enhance our understanding of the inflammatory mechanisms driving gallbladder diseases, and lay the groundwork for future research focused on improving early diagnosis, risk assessment, and treatment outcomes.

## Figures and Tables

**Table 1:** Comparison of parameters between patients with and without adenocarcinoma.

Parameter	Adenocarcinoma	Without Adenocarcinoma	*p* Values
Total bilirubin (mg/dL)	0.55 ± 0.07	1.13 ± 0.70	* 0.047
CA 19-9 (ng/mL)	563.69 ± 52.18	41.96 ± 13.44	* 0.001
CEA (ng/mL)	30.67 ± 2.07	2.81 ± 0.60	* 0.001
AFP (ng/mL)	13.00 ± 0.47	5.21 ± 3.94	* 0.001
Amylase (U/L)	46.00 ± 1.55	190.89 ± 58.13	* 0.001
CA-125 (ng/mL)	578.27 ± 58.43	44.50 ± 13.85	* 0.001

* Statistical significant.

**Table 2:** Laboratory parameters and tumour markers in metaplasia.

Parameter	Metaplasia (*n* = 7)	No Metaplasia (*n* = 83)	Student’s *t*-Test *p*-Value
Hemoglobin (g/dL)	11.50 ± 0.41	13.34 ± 2.03	* 0.019
Creatinine (mg/dL)	1.26 ± 0.21	0.90 ± 0.25	* 0.001
CA19-9 (ng/mL)	58.49 ± 40.58	78.28 ± 20.23	0.778
CEA (ng/mL)	9.20 ± 7.24	4.29 ± 0.97	0.158
AFP (ng/mL)	7.45 ± 5.31	5.58 ± 4.19	0.270
Amylase (U/L)	1273 ± 567	89.19 ± 12.93	* 0.001
CA-125 (ng/mL)	38.38 ± 18.31	83.60 ± 20.72	0.531

* Statistical significant.

**Table 3:** Modified laboratory parameters and tumour markers in patients with chronic cholecystitis.

Parameters	Chronic Cholecystitis (*n* = 44)	No Chronic Cholecystitis (*n* = 36)	Student’s *t*-Test *p*
Hemoglobin, g/dL	12.52 ± 1.43	13.84 ± 2.28	* 0.002
Hematocrit, %	38.27 ± 3.56	40.62 ± 4.42	* 0.007
Leukocytes, n/µL	8014 ± 2942	10,996 ± 5378	* 0.002
AST (TGO), U/L	62.85 ± 15.29	23.52 ± 18.78	* 0.011
ALT (TGP), U/L	102.90 ± 31.53	32.83 ± 25.23	* 0.027
Sodium (Na), mEq/L	140.23 ± 1.55	139.04 ± 3.46	* 0.041
CRP, mg/dL	30.97 ± 10.17	72.25 ± 16.02	* 0.034
CA19-9, ng/mL	30.08 ± 13.49	121.38 ± 33.09	* 0.014
CEA, ng/mL	2.87 ± 5.15	6.39 ± 4.57	0.058
AFP, ng/mL	4.94 ± 3.84	6.49 ± 4.57	0.085
Amylase, U/L	294.61 ± 108.85	72.78 ± 58.31	* 0.041
CA-125, ng/mL	28.27 ± 14.19	129.66 ± 33.60	* 0.008

* Statistical significant.

**Table 4:** Correlation between thrombocytes and tumour markers in adenocarcinoma patients.

Variable 1	Variable 2	Correlation Coefficient	*p*-Value
Thrombocytes	CEA	0.32	0.08
Thrombocytes	CA 19-9	0.56	* 0.02
Thrombocytes	AFP	0.14	0.42
Thrombocytes	CA 125	0.21	0.33
Thrombocytes	CEA	0.30	0.10
Thrombocytes	CA 19-9	0.52	* 0.03
Thrombocytes	AFP	0.18	0.38
Thrombocytes	CA 125	0.23	0.29

* Statistical significant.

**Table 5:** INR correlations with tumour markers in adenocarcinoma patients.

Histopathology Group	Clotting Parameter	Tumour Marker	*p*-Value
Adenocarcinoma	INR	CRP	0.501536
Adenocarcinoma	INR	CEA	* 0.012800
Adenocarcinoma	INR	CA19-9	0.484091
Adenocarcinoma	INR	CA125	0.519248
Adenocarcinoma	INR	AFP	0.936558

* Statistical significant.

**Table 6:** APTT correlations with tumour markers.

Histopathology Group	Clotting Parameter	Tumour Marker	*p*-Value
Adenocarcinoma	aPTT	CRP	0.831
Adenocarcinoma	aPTT	CEA	0.573
Adenocarcinoma	aPTT	CA19-9	0.749
Adenocarcinoma	aPTT	CA125	0.936
Adenocarcinoma	aPTT	AFP	0.894
Benign lesions	aPTT	CRP	0.174
Benign lesions	aPTT	CEA	0.060
Benign lesions	aPTT	CA19-9	0.026
Benign lesions	aPTT	CA125	0.841
Benign lesions	aPTT	AFP	0.644
Metaplasia	aPTT	CRP	0.666
Metaplasia	aPTT	CEA	* 0.001
Metaplasia	aPTT	CA19-9	* 0.001
Metaplasia	aPTT	CA125	0.666
Metaplasia	aPTT	AFP	0.666

* Statistical significant.

**Table 7:** Multivariate analysis of CRP in patients with chronic cholecystitis.

Model	R	R-Squared	Adjusted R-Squared	Std. Error Estimate	F	*p*	Predictors
1	0.196	0.039	0.027	93.65007	3.285	0.074	Age
2	0.349	0.122	0.100	90.05682	7.674	* 0.007	Age, Hb
3	0.410	0.168	0.137	88.21275	4.422	* 0.039	Age, Hb, HT
4	0.418	0.175	0.133	88.38359	0.691	0.408	Age, Hb, HT, amylase
5	0.718	0.515	0.484	68.17896	54.761	* 0.001	Age, Hb, HT, amylase, CA19-9
6	0.751	0.564	0.530	65.07227	8.626	* 0.004	Age, Hb, HT, amylase, CA19-9, CEA
7	0.752	0.565	0.525	65.41154	0.203	0.653	Age, Hb, HT, amylase, CA19-9, CEA, AFP
8	0.752	0.566	0.520	65.78320	0.144	0.706	Age, Hb, HT, amylase, CA19-9, CEA, AFP, CA125

* Statistical significant.

## Data Availability

Data are contained within the article.
